# Fitness as mediator between weight status and dimensions of health-related quality of life

**DOI:** 10.1186/s12955-018-0981-0

**Published:** 2018-07-31

**Authors:** Miguel A. Perez-Sousa, Pedro R. Olivares, Juan A. Escobar-Alvarez, Jose A. Parraça, Narcis Gusi

**Affiliations:** 1 Faculty of Sport Sciences, University of Extremadura, Cáceres. Spain. Av/ Universidad s/n, 10007 Cáceres, Spain; 2 Instituto de actividad física y salud Universidad Autonoma de Chile, Talca, Chile; 3 Sport and Public Services Department, South Essex College. Southend-on-Sea, Southend-on-Sea, England UK; 4 Departamento de Desporto e Saúde, Escola de Ciências e Tecnologia, Universidade de Évora, Évora, Portugal

**Keywords:** Physical fitness, Overweight, Obesity, Mediation analysis, Quality of life

## Abstract

**Background:**

There is evidence that overweight and obesity in children is associated with poor Physical Fitness and consequently lower Health-related Quality of Life (HRQoL). However, this linear-causal relationship between Weight Status → Physical Fitness → HRQoL is not enough to fully understand this phenomenon. Therefore, need to know, through mediation analysis, how operate the Physical Fitness between weight status and HRQoL dimensions.

**Purpose:**

The aim of this study was to determine which HRQoL dimensions are mediated through Physical Fitness in obese (including overweight) and normal weight children. The study also examined the association between Physical Fitness, Body Mass Index (BMI) and HRQoL.

**Methods:**

A total of 233 overweight/obese children and 105 normal-weight children participated in the study. Children were recruited from public educational centers and a public weight loss program. BMI, Physical Fitness (upper limb, central body and lower limb strength; agility and range of motion) and HRQoL (PedsQL and VAS) were measured. Simple mediation analyses by gender, through ﻿PROCESS macro developed by Preacher and Hayes, were performed in order to analyze whether Physical Fitness computed as z-score, is a mediator in the relation between weight status (normal weight or overweight/obesity) and HRQoL dimensions. \itionally, unequal-variances t statistics were executed to know differences in BMI, Physical Fitness components and HRQoL dimensions between groups, and correlations to know the associations between weight status, Physical Fitness z-score and HRQoL.

**Results:**

Our results, indicated association between the Physical Fitness z-score and HRQoL dimensions in overweight/obese children. Regarding to mediation analysis, the results showed that the negative association between overweight/obesity and HRQoL is softened by the level of Physical Fitness. Therefore Physical Fitness is a mediator in the relationship between overweight/obesity children and the most of dimensions of HRQoL, except the School functioning in boys and the School and Emotional functioning in girls.

**Conclusions:**

The negative effect of overweight or obesity on HRQoL inn children, is mitigated by Physical Fitness. Consequently, the Physical Fitness is a mediator on HRQoL in most dimensions, especially daily living, in schoolchildren.

**Electronic supplementary material:**

The online version of this article (10.1186/s12955-018-0981-0) contains supplementary material, which is available to authorized users.

## Background

The prevalence of overweight and obesity in children and adolescents has continuously been increasing in Spain [1] and most European countries since the 1990s [2]. Thus, maintaining public health strategies is necessary to reduce this phenomenon since overweight and obesity have been associated with a number of comorbidities related to physical and psychological health [3–5] such as high risk of hypertension, hyperinsulinemia, dyslipidemia, and type 2 diabetes [6]. In addition, children and adolescents who are overweight or obese have a poor physical fitness (PF) level, e.g. in muscular strength [7, 8] and cardiorespiratory endurance [9, 10], and consequently show low motor skill and coordination performance [11]. In this binomial, physical activity (PA) is a key determinant because low PA levels accelerate the detriment of PF [11]. Consequently, a high body mass index (BMI), and low PA and PF levels have been closely related to poor health-related quality of life (HRQoL) [12, 13] and this impact is directly proportional to weight status [14]. Therefore, the description of the simple and direct relationship between BMI-PF-HRQoL is not enough to fully understand and obtain evidence about these causal phenomena.

Statistical mediation analysis allows us to understand how an independent variable *“X”* affects a dependent variable *“Y”* through the indirect effect of the mediating variable *“M”* [15]. For instance, mediation analysis could identify if PF level do and do not mediate these children’s quality of life dimensions. This knowledge could help to adjust the program to the PF level or BMI of children by establishing adequate subgroups.

The relationship between PF and HRQoL in overweight or obese children has been studied extensively in recent years, but without reporting a comprehensive battery of fitness tests and its direct or indirect influence as a mediator in HRQoL dimensions. Some previous works used mediation analysis to study the relationships between amount of body fat, PF and academic performance in children [16–18]. However, these studies did not mention the role of the mediating variable according to the confidence interval suggested by Hayes [15]. Therefore, additional research is required to delve into the knowledge about the relationships between multi-attribute physical fitness and HRQoL.

The purpose of this study was to analyze how PF affects the link between overweight/obesity versus normal weight and HRQoL dimensions.

## Methods

### Study design

This cross-sectional study is the baseline of the public health program “Exercise Looks After You” organized by the University of Extremadura and funded by Health & Dependence Department and Young & Sports Department of Extremadura Government (Spain). It arose to improve the HRQoL of obese and overweight children through learning and practicing basic fitness, sport and social skills to empower them.

A trainer specializing in PA in children conducted the present study in the region of Extremadura with children and adolescents who completed a training course consisting of three weekly sessions lasting an hour each, whose purpose was to learn games and physical group activities. The same assistant from the research group conducted all tests at the fitness center.

### Participants

Two hundred thrity-three overweight or obese children ranging in age from 6 to 14 (M 9.2, SD = 2.0) and 105 with normal weight children ranging in age from 7 to 14 (M 10.8, SD = 1.8) participated in the study. The non-overweight or obese population was recruited from two primary and secondary schools and the obese population was referred by pediatricians in the Spanish National Health System. To obtain a significant sample of obese children, 6 sport technicians from the public health program completed an advertising campaign at primary care centers, schools, sport clubs and neighborhood communities promoting participation in the study through information stands which distributed posters and flyers to parents, teachers, social workers, nurses and physicians. The campaign included the following: a) support of the study from the regional government and the university, b) participants did not pay any fees, c) participants received an individual health-related fitness report after taking part in the test battery and completing the HRQoL questionnaire, d) participants would undergo a short medical examination to ensure that they could do PA.

The participants had to comply with the following inclusion criteria: be overweight or obese; have the ability to move themselves; not suffer from any disease that made it impossible to do PA; sign the informed consent themselves or have their parents do so; understand each item from the questionnaire and perform the test battery safely.

This study was approved by the Committee on Biomedical Ethics of the University of Extremadura (n°98/2007) and following the precepts of the Declaration of Helsinki [19].

### Measurements

A set of questionnaires including demographic questions, weekly level of PA and HRQoL was administered as well as a battery of fitness tests.

#### Anthropometric data

Body mass index (BMI): body weight was measured to the nearest 0.1 kg using a Tanita SC-330, (Tanita Corp., Japan). Height was estimated with an aluminum stadiometer (Seca 713 model, Postfach, Germany) to the nearest 1 mm. BMI was calculated as body weight divided by the square height (kg/m2). The BMI variable was categorized into normal weight, overweight and obese according to the indications of Cole et al. [20].

Health-related quality of life.

HRQoL was assessed using the PedsQL Generic Core Scale and the Visual Analogue Scale (VAS) from the EQ-5D-Y [21].

The PedsQL Generic Core Scale [22] is an instrument with 23 Likert scale items, grouped into four sub-scales: Physical Functioning (8 items), Emotional Functioning (5 items), Social Functioning (5 items) and School Functioning (5 items). Applying the PedsQL score, we can obtain the following 5 dimensions: 1) the Physical Health Summary Score equal to the Physical Functioning subscale; 2) the Psychosocial Health Summary Score calculated by the sum of Emotional, Social and School Functioning divided by the 15 items; 3) the Total Score is calculated as the sum of 23 items divided by the number of items answered.

The VAS from EQ-5D-Y [21] is a continuous line between two endpoints where the respondent has to rate his or her own health status, with 0 being the worst and 100 the best imaginable health status.

#### Fitness

Each participant completed a battery of fitness tests performed in a gymnasium with a temperature and humidity between 19°-22 °C and 40–60%, respectively. The following fitness tests were measured:

- Upper body strength was measured through a handgrip test using a hand dynamometer (TKK 5401 model, Tokyo, Japan) following standardized protocol [23, 24]. Two measurements were taken for each hand and the sum of the best for each hand was recorded as the score for analysis.

- Lower body strength was assessed using the countermovement vertical jump (CMJ) and standing long jump (SLJ). The CMJ was conducted using a contact platform connected to an electronic timer (Chronojump Bosco system, Barcelona, Spain) [25]. The CMJ was done with the subjects starting in a standing position with feet shoulder-width apart and hands placed on the pelvic girth [26]. The SLJ was done with the subjects standing behind a line marked on the ground with feet slightly apart and jumping as far forward as possible. The distance was measured in cm from the starting line to the nearest point of contact on the landing (back of the heels) [24].

- Core body strength was assessed using the curl up test. This test consists of completing the maximum number of repetitions of sit ups (up and back) possible in 30s. Only one trial for this test was done.

- Range of Movement (ROM): the sit and reach test was performed to assess the ROM in pelvic and lumbar bending. The best of two attempts was used for the statistical analysis [27].

- Agility: the 4 × 10-m shuttle run was used to assess agility and speed. The subjects ran between two parallel lines marked on the ground (10 m) as fast as possible crossing each line with both feet every time. The stopwatch was stopped when the subject crossed the line for the fourth time. Two trials were done and the best result was recorded [24, 28].

### Procedure

The participants were instructed to wear sportswear and sneakers and not eat heavy food 2 h before as well as not to perform vigorous PA 48 h before the assessment. Before starting the fitness test battery, the children performed an 8-min warm-up including running and joint mobility exercises. The fitness test battery was carried out in the following order: hand grip, CMJ, sit and reach, curl up, SLJ and shuttle run.

### Statistics

A descriptive analysis using mean ± standard deviation for the continuous variables and frequency distribution for categorical variables was used to obtain the characteristics of the sample. The z-scores adjusted by age and gender for fitness test were calculated obtaining a variable for each test except the SLJ and CMJ that were combined (by means of z-scores in these tests) in one variable called “lower limb strength”. To obtain a global fitness index, a new variable was calculated using the mean values of all z-scores (except the SLJ and CMJ which were included using the previously calculated lower limb strength variable).

﻿Statistical normality of the variables was tested using both graphical (normal probability plot) and statistical procedures (Kolmogorov–Smirnov test).

The Mann-Whitney U test was performed to analyze PF components and HRQoL differences between the normal weight and overweight-obese groups. Spearman Rho correlation coefficients were calculated to obtain the relationships between HRQoL dimensions, anthropometric measurements and PF.

Simple mediation analyses were conducted using ordinary least squares path analysis to examine whether the relation between weight status and HRQoL was mediated by PF using the PROCESS macro for SPSS (IBM, Chicago, IL, USA) [29]. The models were composed by weight status that according to BMI values, was created a dummy variable (normal weight Vs overweight/obese) as an independent variable, the HRQoL (PedsQL dimensions and EQ-5D-Y VAS) as dependent variables, and the global z-score PF index as the mediating variable. Mediation hypotheses were tested using the bias-corrected bootstrap method with 10,000 samples to calculate confidence intervals (95%). Indirect effect was considered significant when the confidence interval did not contain zero [15].

## Results

Table 1 presents the major characteristics of the sample. In the overweight/obese group, 32.2% of participants did not perform any hours of PA per week and 20.2% performed more than three, while in the normal weight group, 36.2% of participants performed three or more hours of PA per week and only 1.0% did not perform any hours of PA per week. In both groups, boys were more physically active than girls.

**Table 1:** Participant characteristics by BMI group and gender

	Overweight (*n* = 91) / Obese (*n* = 142)			Normal weight (*n* = 105)		
	boys	girls	All	Boys	girls	All
	125 (53.6)	108 (46.4)	233 (68.9)	49 (46.7)	56 (53.3)	105 (31.1)
Physical activity Level						
0 h/week	32 (25.6)	43 (39.8)	75 (32.2)	0 (0.0)	1 (1.8)	1 (1.0)
< 3 h/week	59 (47.2)	48 (44.4)	107 (45.9)	25 (51.0)	42 (75.0)	66 (62.9)
> 3 h/week	33 (26.4)	14 (13.0)	47 (20.2)	24 (49.0)	13 (23.2)	38 (36.2)
BMI	24.5 (4.0)	24.3 (3.4)	24.4 (3.7)	18.2 (2.0)	18.6 (1.8)	18.4 (1.9)

^a^*note*: date expresed by mean and SD

*BMI* body mass index (kg/m^2^)

Table 2 shows the outcomes obtained in the fitness tests by total sample and split by gender. Normal weight group (boys and girls) performed better in most fitness tests (*p* < 0.05) than overweight/obese group, except the upper body strength in boys, in which overweight/obese children have the same performance. In addition, in Table 3, it is noted the HRQoL (PedsQL dimensions and EQ-5D-Y VAS) by BMI group and gender. According results from U Mann-Whitney there are differences between overweight/obese and normal weight children in most dimensions of PedsQL excluding the Social functioning both in boys as in the total sample. In addition, it can also be observed that in overweight /obese and normal weight girls there are no significant differences in the EQ-5D-Y VAS.

**Table 2:** Physical Fitness characteristics and differences by BMI group and gender

	Boys	Girls	All
Upper body strength (kg*cm2)			
Normal weight	39.2 (6.1)	**36.9 (5.0)**	**38.0 (5.6)**
Obese / overweight	39.3 (11.5)	**32.7 (10.0)**	**36.2 (11.3)**
Central body strength (rep)			
Normal weight	**31.6 (6.5)**	**24.2 (5.4)**	**27.7 (7.0)**
Obese / overweight	**24.8 (17.4)**	**17.8 (11.7)**	**21.5 (15.4)**
Lower body strength			
CMJ (cm)			
Normal weight	**27.0 (5.0)**	**21.3 (4.2)**	**24.0 (5.4)**
Obese / overweight	**23.3 (5.6)**	**19.5 (5.2)**	**21.5 (5.7)**
SLJ (cm)			
Normal weight	**133.4 (11.4)**	**104.5 (10.6)**	**118.0 (18.1)**
Obese / overweight	**110.7 (26.9)**	**97.1 (18.5)**	**104.4 (24.3)**
Agility (sec)			
Normal weight	**13.0 (0.8)**	**14.9 (2.2)**	**14.1 (1.9)**
Obese / overweight	**14.2 (2.2)**	**15.2 (1.4)**	**14.7 (1.9)**
ROM (seat and reach) (cm)			
Normal weight	**25.2 (8.2)**	**28.7 (8.0)**	**27.1 (8.2)**
Obese / overweight	**20.6 (9.2)**	**22.5 (8.8)**	**21.5 (9.1)**

*Note*: data expressed by mean (SD); U Mann Withney test to compare differences between BMI groups; bold type = *p* < .05

**Table 3:** Health-related Quality of Life characteristics and differences by BMI group and gender

PedsQL Dimensions	Boys	Girls	All
Physical			
Normal weight	**91.3 (9.2)**	**93.3 (6.7)**	**92.4 (8.0)**
Obese / overweight	**82.4 (15.1)**	**80.4 (14.7)**	**81.4 (14.9)**
Emotional			
Normal weight	**86.3 (13.7)**	**88.5 (12.3)**	**87.5 (13.0)**
Obese / overweight	**77.1 (20.7)**	**80.3 (17.4)**	**78.6 (19.2)**
Social			
Normal weight	85.9 (14.4)	**91.0 (9.9)**	88.6 (12.4)
Obese / overweight	85.8 (17.1)	**83.3 (18.5)**	84.6 (17.8)
School			
Normal weight	**90.2 (12.2)**	**91.8 (9.9)**	**91.0 (11.1)**
Obese / overweight	**82.6 (17.1)**	**80.6 (15.9)**	**81.7 (16.6)**
Psychosocial			
Normal weight	**87.4 (10.6)**	**90.4 (8.0)**	**89.0 (9.4)**
Obese / overweight	**81.8 (14.8)**	**81.4 (13.8)**	**81.6 (14.3)**
Total Health			
Normal weight	**88.8 (8.7)**	**91.4 (7.1)**	**90.1 (7.9)**
Obese / overweight	**82.0 (13.4)**	**81.0 (12.1)**	**81.6 (12.8)**
EQ-5D-Y VAS			
Normal weight	**89.7 (14.8)**	88.6 (13.8)	**89.1 (14.2)**
Obese / overweight	**84.3 (15.4)**	83.8 (16.0)	**84.0 (15.6)**

*Note* data expressed by mean (SD).

U Mann-Whitney test to compare differences between normal weight vs obese/overweight

bold type = *p* < 0.05

Correlation coefficients between PF z-scores, anthropometric measurements and HRQoL dimensions are shown in Additional file 1: Table S1. Statistically significant correlations were found in anthropometric measurements, VAS and PedsQL dimensions except Social and School Functioning in overweight/obese boys, and no significant correlations were found in Emotional, School and VAS in overweight/obese girls. However, no significant correlations were found in the normal weight group for any variable, except in BMI and School Functioning in girls.

**Fig. 1:**
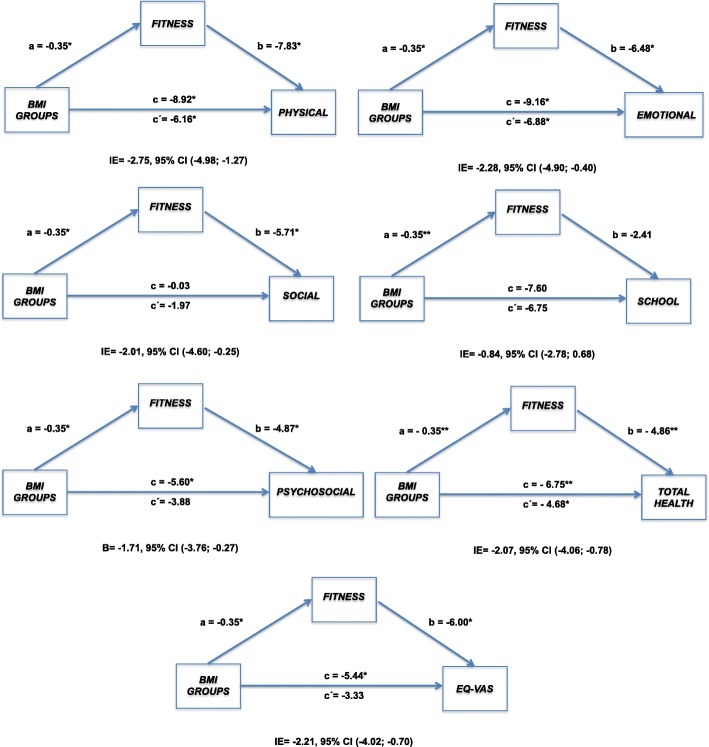
Mediation analysis (boys) of weight condition (normo-weight Vs overweight/obese) on HRQoL through Physical Fitness with 10,000 bootstrap. The negative association between weight condition and HRQoL, is attenuated by level of Physical Fitness.. The indirect effect is statistically significant at the 95% confidence interval (CI) when the CI does not include

**Fig. 2:**
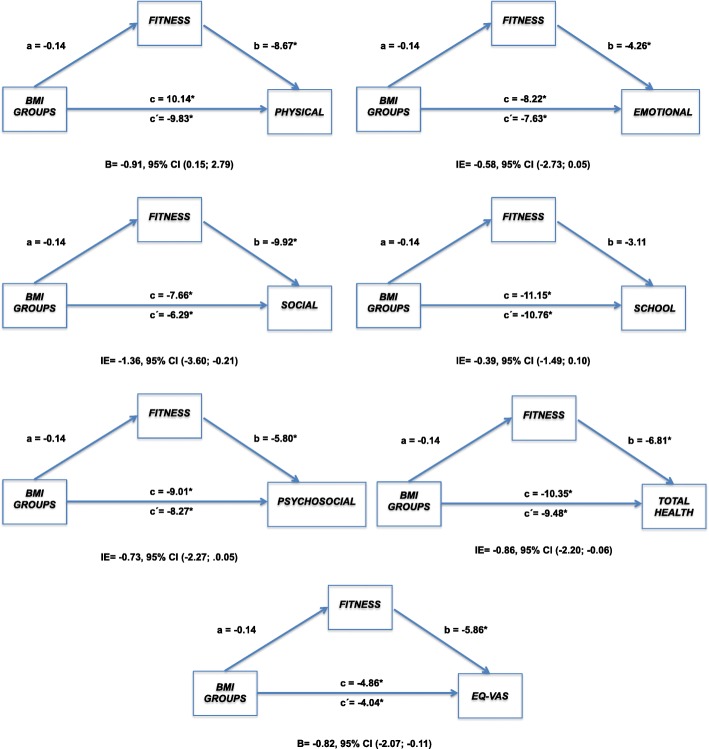
Mediation analysis (gilrs) of weight condition (normo-weight Vs overweight/obese) on HRQoL through Physical Fitness with 10,000 bootstrap. The negative association between weight condition and HRQoL, is attenuated by level of Physical Fitness.. The indirect effect is statistically significant at the 95% confidence interval (CI) when the CI does not include

Figures 1 and 2 provide the mediation analysis for the effect of overweight/obese or normal weight status on PedsQL dimensions and EuroQol VAS through global z-score PF index. In the regression model, were included age and PA level as covariates, however these variables not had significant effect on the regression, consequently were excluded.

With regard to mediation analysis, results showed that PF acts as a mediator in the relationship between overweight/obese or normal weight status and most HRQoL dimensions in both boys and girls. For boys, the only dimension in which PF did not mediate this relationship was School Functioning, and for girls the School and Emotional Functioning dimensions were not significant.

## Discussion

Major novelties of the current study were: i) the mediation analysis that led to identifying the key PF split by gender that greatly influence the different perception of HRQoL and health (VAS) that overweight and obese children showed compared to normal-weight participants; ii) an improved multi-attributed PF global index.

Firstly, the consistency of data with previous studies was tested. The descriptive and variances t statistical analysis between BMI groups reveal significant differences in all PF components, except in upper body strength of boys, which performance was very similar. Previous studies also found similar differences between conditions in PF performance [30, 31]. Additionally, the overweight/obese group showed a lower HRQoL, and significant differences were found in all HRQoL dimensions and VAS between genders and the total sample [32, 33]. Therefore, the data from the current study is consistent with previous literature.

A global PF index was calculated based on z-scores from all PF tests as a standardized average to be used in the correlations and mediation analyses. This index allows a global analysis of the relationship between FP with anthropometric measurements and HRQOL dimensions, and constitutes a more representative value of an individual’s fitness than each fitness test alone. This amelioration of the PF index by including more PF attributes is relevant because the perception of health-related quality of life using VAS and the PedQSL are also global and generic outcomes. Concerning the correlations between PF, anthropometrics, HRQoL dimensions and VAS, a high association between PF and anthropometrics; Physical, Emotional, and Psychosocial Functioning; Total Health and VAS can be observed in obese or overweight boys. These findings are consistent with previous studies in overweight and obese children, which reflects significant associations between cardiorespiratory and musculoskeletal fitness and several HRQoL dimensions such as Psychosocial, Physical and Social Functioning, [13, 34, 35]. A similar association was found in overweight or obese girls, particularly in the Social dimension which significantly correlates with PF. These findings reflect that PF level is more associated with the self-perception of social confidence in overweight/obese girls, thus physical conditioning programs require a gender perspective which, in addition to training in the different fitness components, promotes social skills to improve overweight status in society (e.g. walking around with friends and socializing). Interestingly, the VAS (best imaginable health today) does not correlate with PF and it is interesting to note that the perception metrics in girls differed from the Total Health Score extracted from the PedsQL. It could be partly attributable to the different design of the instruments and index that probably led to a different concept perception. While the global index from the PedsQL is forced to be composed by several dimensions, VAS is a global perception of health that girls could perceive as more biological. Nevertheless, it requires further methodological research on this self-perception.

Additionally, our results showed that the PF has a mediating role in HRQoL in obese/overweight or normal weight status in some dimensions of PedsQL.

Concretely, in boys the PF is a mediator between overweight/obese status and HRQoL (Physical, Emotional, Social, and Psychosocial Functioning and Total Health in the PedsQL and EuroQol VAS). However, it does not have a mediating role in the School dimension. Therefore, it seems that the PF not only has a mediating effect on the Physical dimension, Total Health and self-perception of health, but also over dimensions more closely related to psychosocial and peer relationships.

On the other hand, in girls the Physical, Social, and Psychosocial Functioning and Total Health in PedsQL dimensions and VAS are mediated by PF. But PF does not have a mediating effect on Emotional and School Functioning, thus in girls these dimensions are not going to be influenced by the PF status unless the PA program is composed of group activities where peer relationships are a goal of achievement. Overall, the psychosocial and social dimensions were strongly influenced by PF in both genders, reinforcing the great relevance found in correlations in girls and discussed above. To our knowledge, there are no previous studies analyzing the mediating role of fitness between overweight/obesity and HRQoL. In the current literature, there are studies that analyze the mediating role of obesity status between cardiorespiratory fitness and metabolic risk [36], blood pressure [17] and the role of cardiorespiratory fitness and muscular strength within the relationship between fat content and academic achievement [18]. In particular, our study applied a fitness test battery to assess fitness components involved in daily life activities such as coordination-agility, range of motion or whole-body strength. In this way, it provides complementary and better transferable knowledge about which of the HRQoL dimensions are mediated by PF in overweight/obese status in daily living. In addition, our findings could also help improve the response based on the improvement of the FP, the cardiometabolic profile, which interacts with HRQoL, as indicated in recent studies [37].

### Applications

Taking into account the mediation models obtained, a physical exercise protocol to promote HRQOL in children affected by obesity and overweight would be more effective if a greater percentage of the time included several activities aimed at improving muscular strength, agility and ROM. All this, framed in group activities striving for empowerment in PA and the improvement of psychosocial skills to manage the condition and PF level in their relationships outside of school.

### Limitations

The cross-sectional design does not allow us to make cause and effect inferences. More mediation studies analyzing the relationships between obesity, fitness and HRQoL are needed to provide information on this issue, in order to establish evidence about not only the effects but also how to respond to the how and why of these relationships in overweight/obese populations or another health conditions. The generic concept of VAS health used could lead to underestimating the relevance of psychosocial perception in health. However, the current study also includes a multidimensional instrument that complements this information, reinforcing the relevance of other dimensions. Further studies to weigh or evaluate the relative relevance of the different dimensions on generic health perception in children are warranted because some usual methods do not work equally well in adults and in children [38]. Another limitation that should be account are the contextual factors, since the children proceeded of several city of Extremadura.

## Conclusions

The negative effect of overweight or obesity on HRQoL in children, is softened by physical fitness. As consequence, physical fitness plays a major role as a mediator in the relationship between weight status and HRQoL in some of the dimensions, especially daily living, and this tells us that future weight loss programs should combine the improvement of physical fitness status and the empowerment of children in sports practice as a social task, understanding this as a more complete system where more diverse tasks should be done seeking.

## Additional file

Additional file 1: **Table S1.** Correlation coefficients between Physical Fitness, BMI and HRQoL. (DOCX 21 kb)

## Abbreviations

BMI: Body mass index
CMJ: Countermovement vertical jump
HRQoL: Health-related quality of life
PA: Physical activity
PF: Physical fitness
ROM: Range of movement
SLJ: Standing long jump
VAS: Visual analogue scale
