# Supplementary material for: Fitness as mediator between weight status and dimensions of health-related quality of life
Source: Health Qual Life Outcomes. 2018 Jul 31;16:155. doi: 10.1186/s12955-018-0981-0 (PMC6069830; doi:10.1186/s12955-018-0981-0)
Supplement: Supplementary file 1 — Table S1. Correlation coefficients between Physical Fitness, BMI and HRQoL. (DOCX 21 kb) [file 12955_2018_981_MOESM1_ESM.docx]

| **Additional file 1: Table S1. Correlation coefficients between Physical Fitness, BMI and HRQoL.** | | | | |
| --- | --- | --- | --- | --- |
|  | **boys** | | **girls** | |
|  | **overweight / obesity** | **normal weight** | **overweight / obesity** | **normal weight** |
| **BMI** | -.538** | .111 | -.249** | -.282* |
| **PedsQL Physical** | .342** | -.046 | .404** | -.057 |
| **PedsQL Emotional** | .237** | .170 | .158 | -.125 |
| **PedsQL Social** | .153 | .013 | .376** | -.170 |
| **PedsQL School** | .028 | .073 | .167 | -.357** |
| **PedsQL Psychosocial** | .196* | .131 | .295** | -.253 |
| **PedsQL Total Health** | .305** | .082 | .402** | -.225 |
| **EQ-5D-Y VAS** | .262** | -.223 | .142 | .202 |
| Spearman´s Rho correlation coefficients between Physical Fitness (Z-Score) and Anthropometrics measures, PedsQL dimensions and VAS * p < 0.05; ** p < 0.01 | | | | |
